# Celebrity worship and cognitive skills revisited: applying Cattell’s two-factor theory of intelligence in a cross-sectional study

**DOI:** 10.1186/s40359-021-00679-3

**Published:** 2021-11-08

**Authors:** Lynn E. McCutcheon, Ágnes Zsila, Zsolt Demetrovics

**Affiliations:** 1 North American Journal of Psychology, 240 Harbor Drive, Winter Garden, FL 34787 USA; 2 Institute of Psychology, Pázmány Péter Catholic University, Mikszáth Kálmán tér 1., Budapest, 1088 Hungary; 3 Centre of Excellence in Responsible Gaming, University of Gibraltar, Gibraltar, Gibraltar; 4 Institute of Psychology, ELTE Eötvös Loránd University, Kazinczy u. 23-27, Budapest, 1075 Hungary

**Keywords:** Celebrity worship, Cognitive skills, Intelligence, Material wealth, Self-esteem

## Abstract

**Background:**

Almost two decades of research produced mixed findings on the relationship between celebrity worship and cognitive skills. Several studies demonstrated that cognitive performance slightly decreases with higher levels of celebrity worship, while other studies found no association between these constructs. This study has two aims: (1) to extend previous research on the association between celebrity worship and cognitive skills by applying the two-factor theory of intelligence by Cattell on a relatively large sample of Hungarian adults, and (2) to investigate the explanatory power of celebrity worship and other relevant variables in cognitive performance.

**Methods:**

A cross-sectional study design was used. Applying an online survey, a total of 1763 Hungarian adults (66.42% male, *M*_*age*_ = 37.22 years, *SD* = 11.38) completed two intelligence subtests designed to measure ability in vocabulary (Vocabulary Test) and digit symbol (Short Digit Symbol Test). Participants also completed the Celebrity Attitude Scale and the Rosenberg Self-esteem Scale. Subjective material wealth, current family income and general sociodemographics were also reported by participants.

**Results:**

Linear regression models indicated that celebrity worship was associated with lower performance on the cognitive tests even after controlling for demographic variables, material wealth and self-esteem, although the explanatory power was limited.

**Conclusions:**

These findings suggest that there is a direct association between celebrity worship and poorer performance on the cognitive tests that cannot be accounted for by demographic and socioeconomic factors.

**Supplementary Information:**

The online version contains supplementary material available at 10.1186/s40359-021-00679-3.

## Introduction

It has been about two decades since a study first showed that those who scored high on a measure of attraction to one’s favorite celebrity also tended to score low on measures of cognitive skills [1]. Since then, research has produced mixed findings on the relationship between celebrity worship and cognitive skills. Several studies demonstrated that cognitive performance slightly decreases with higher levels of celebrity worship [2–4], although some studies found only limited support for the association between these constructs [5, 6]. An early study by Martin et al. [2] proposed that celebrity worship may interfere with the information processing of individuals who are more prone to get absorbed in the personal details of a celebrity. Celebrity worship, as an excessive behavior, was also associated with several behavioral addictions (e.g., problematic Internet and social media use [7, 8], compulsive buying [9], gambling addiction [10]), and these problematic behaviors are known to have negative effects on school/work performance, social relationships and cognitive functioning [11]. Therefore, it can be expected that excessive involvement with an admired celebrity may interfere with cognitive performance due to the limited ability to focus on other things than the celebrity. Although some studies supported this notion by revealing an association between celebrity worship and poorer cognitive skills [2–4], these studies were conducted on a sample of less than 200 college students from the UK and USA, focused only on a few cognitive skills without a theoretical frame, and did not explore the role of socioeconomic factors (e.g., income, subjective wealth, education) when investigating the contribution of celebrity worship to poorer performance on cognitive tests. Therefore, there is a lack of information regarding the possible contribution of socioeconomic factors to cognitive performance besides celebrity worship. This study endeavors to extend previous research on the direct association between celebrity worship and cognitive performance by using the two-factor theory of intelligence by Cattell [12, 13]. The present research was conducted on a relatively large sample of Hungarian adults (*N* = 1763), which allow the exploration of this association in a culturally different context. This study also aims to draw a clearer picture of the explanatory power of celebrity worship and other relevant socioeconomic factors in various cognitive skills. The investigation of these associations could possibly increase our understanding of the diversity of cognitive performance among fans. Specifically, we measured the relationship between fluid intelligence, crystallized intelligence, and the admiration for one’s favorite celebrity, controlling for extraneous variables that might explain earlier findings. The main control variables in our research were measures of self-esteem, current family income, and perceived relative material wealth.

### Celebrity worship

Celebrity worship has been defined as an increased admiration towards a famous person, which sometimes manifests in an excessive interest in the life of a celebrity (see [14, 15]). The *Celebrity Attitude Scale* (CAS) was developed in order to have a reliable and valid way to measure excessive devotion to celebrities. Factor analysis has revealed three increasingly more extreme sets of attitudes and behaviors associated with celebrity worship. A low level of celebrity worship, dubbed *Entertainment-Social,* has generally been shown to be benign [16]. The second level, labeled *Intense-Personal*, reflects individuals’ compulsive feelings about the celebrity. The most extreme expression of celebrity worship is labeled *Borderline-Pathological.* This third level is believed to reflect an individual’s borderline pathological attitudes and behaviors toward a favorite celebrity. In most studies, the second and third levels were associated with problematic behavior [17, 18].

### Cognitive skills

There are several studies that have found modest relationships between scores on the CAS and various measures of cognitive ability. Specifically, these relationships collectively indicate that persons who tend to excessively admire their favorite celebrities also tend to score lower on measures of cognitive ability (see [19] for a review).

A study of American college students yielded several significant correlates of CAS scores and measures of cognitive skills. Specifically, CAS correlated negatively with a measure of creativity called the *Remote Associates Test* [20], a test of information similar to those found on IQ tests, a test of critical thinking ability, and a “Squares” problem, in which participants had to determine the total number of squares embedded within a very large square. Correlation coefficients ranged between − 0.39 and − 0.31, and two of the three were significant at the 0.001 level. Two other measures, an arithmetic test and the *Need for Cognition* scale [21], also correlated negatively with CAS scores and barely missed significance [1]. A followup study attempted to generalize these results to other cognitive measures with limited success. Scores on an *Advanced Reasoning Skills* test correlated negatively with all three subscales of the CAS, but was significant on only one of them. Scores on the *Intellectual Flexibility Scale* [22] correlated negatively but weakly with only one of the three CAS subscales. In addition, an attempt to determine why celebrity worship is linked to cognitive ability failed to produce meaningful results [5].

Using a measure of cognitive flexibility different from that used by McCutcheon et al. [5], researchers found significant negative correlations with two of the three CAS subscales [2]. A study of three analytic thinking ability measures formed a pattern of relationships with the three subscales of the CAS. Eight of the nine resulting correlation coefficients were negative, and four of them were significant at the 0.01 level [3]. A recent study found that the correlation between total CAS scores and a vocabulary test, an environmental knowledge test, and a test of knowledge of nature-related words ranged from − 0.30 to − 0.39, all significant at the 0.001 level [23].

Based on these studies, it is apparent that high scores on the CAS are associated with lower scores on various measures of cognitive ability. However, at least two things about this relationship are not so clear. For one, what are the underlying causes of this relationship? Is it possible that some third variable is responsible for the link between cognitive ability and celebrity admiration? This study calls for plans to control for some correlates of cognitive ability, (i.e. self-esteem, current family income, material wealth, highest level of education) in an attempt to identify one or more variables that might be responsible for the link between cognitive skills and celebrity worship.

Furthermore, no previous study has attempted to fit the link between cognitive ability and celebrity admiration into a theoretical framework. We are adopting the two-factor theory of intelligence developed by Cattell. According to Cattell, intelligence can be conceptualized as broadly consisting of fluid and crystallized intelligence [12, 13]. The former is the ability to reason, see patterns, analyze, and solve new problems, without using much previously obtained knowledge. It is considered to be relatively culture free. The critical thinking test used by McCutcheon et al. [1] and the *Advanced Reasoning Skills* test [5] both measure fluid intelligence.

In the present study we used items from the *Short Digit Symbol Test* (SDST) as a measure of fluid intelligence. A similar digit symbol test has been shown to load 0.63 on a performance/fluid factor of intelligence but only 0.17 on a verbal/crystallized factor [24]. Crystallized intelligence, on the other hand, is the ability to use one’s skills and knowledge obtained in the past. The vocabulary test used by Aruguete et al. [23] and the information test used by McCutcheon et al. [1] exemplify attempts to measure crystallized intelligence. In the present study we used a vocabulary test similar to that used by Aruguete and colleagues, but adapted for Hungarians. Vocabulary knowledge is highly related to general intelligence [25], and is widely considered to be a strong measure of crystallized intelligence [26].

Based on our literature review, the present study aims to extend previous research on the association of celebrity worship with cognitive skills by applying Cattell’s two-factor theory of intelligence. The relatively large sample of Hungarian adults allows for the testing of this association in a culturally diverse background compared to previous studies that have used mostly student samples from the UK and the USA. Furthermore, this study aims to explore the contribution of celebrity worship and other relevant factors (e.g., demographic characteristics, material wealth, self-esteem) to cognitive performance. The present investigation also addresses the question of whether celebrity worship has a unique contribution to cognitive performance on tests measuring crystallized and fluid intelligence. The exploration of these associations could provide with a more nuanced picture of the nature of the association between celebrity worship and cognitive ability.

### Hypothesis

#### H1

Performance on the vocabulary test (crystallized intelligence), the short digit symbol test (fluid intelligence) and the combination of both will decrease with increasing levels of celebrity worship.

#### H2

There will be a negative relationship between scores on the vocabulary test, the short digit symbol test and the combination of both and celebrity worship after controlling for gender, age, educational level, current family income, current and childhood material wealth and self-esteem.

#### RQ1

Which are the most powerful predictors of cognitive performance on the vocabulary and the short digit symbol tests?

## Methods

### Participants and procedure

The participants were recruited from a popular Hungarian news website (444.hu). Participants were invited to complete an online questionnaire focusing on attitudes towards celebrities and cognitive/mental status. Participation was voluntary and anonymity was provided for the respondents. Informed consent was obtained from all participants. The research was conducted with the approval of the research team’s university and was carried out following the principles of the Declaration of Helsinki.

A total of 1763 Hungarian adults (66.42% male, *M*_*age*_ = 37.22 years, *SD* = 11.38, age ranged from 18 to 79 years) completed the online survey. The majority of participants reported having a college degree or higher (*n* = 1244; 70.56%), while another considerable proportion of them reported having a secondary school certification (*n* = 479; 28.19%), and only 1.25% (*n* = 22) completed eight or less classes at primary school. Nearly one-third of participants reported having 301,000–600,000 HUF (about 1005–2003 USD) as a monthly household income after taxes (*n* = 554; 31.42%), one-quarter reported having 101,000–300,000 HUF (about 337–1002 USD) (*n* = 451; 25.58%), and only a small minority reported having less than 100,000 HUF (about 334 USD) (*n* = 60; 3.40%). Another considerable proportion of participants reported having 601,000–1,000,000 HUF (about 2006–3338 USD) as a monthly household income after taxes (*n* = 314; 17.81%), while only a small proportion of participants reported having more than 1,000,000 HUF (about 3338 USD) monthly.

### Measures

*Celebrity Attitude Scale* (CAS). The 23-item version of the CAS has good psychometric properties [14, 27–30]. The response format for the CAS is a 5-point Likert scale with “strongly disagree” = 1 and “strongly agree” = 5. High scores suggest a strong attraction to one’s favorite celebrity.

The CAS consists of three subscales. *Entertainment-Social* (ES) is reflected in agreement with items like “My friends and I like to discuss what my favorite celebrity has done,” A second level of celebrity worship is characterized by more *Intense-Personal* (IP) feelings, defined by items like “I have frequent thoughts about my celebrity, even when I don’t want to.” The third level, labeled *Borderline-Pathological* (BP), is shown in items like: “If I were lucky enough to meet my favorite celebrity, and he/she asked me to do something illegal as a favor I would probably do it.” Across several studies total scale Cronbach alphas ranged from 0.84 to 0.94 [15]. Cronbach alpha in the present study was 0.91 for the total scale, 0.84 for the Entertainment–Social subscale, 0.83 for the Intense–Personal subscale, and 0.55 for the Borderline–Pathological subscale.

The Hungarian version of the *Rosenberg Self-Esteem Scale* (RSES-HU; [31, 32]) consists of 10 items, examples of which are “On the whole, I am satisfied with myself,” and “At times I think I am no good at all” (reverse scored). “Strongly Disagree” is equal to 1, and “Strongly Agree” is equal to 4. High scores suggest a person who has high self-esteem. The RSES has been widely accepted in the scientific community [33]. Cronbach alpha in the present study was 0.90 for the total scale.

*Current Family Income* is a self-reported estimated measure of the amount of monthly household income after taxes for the family. The question and response categories were derived from the National Survey on Addiction Problems in Hungary (OLAAP) [34]. For the data analysis, this variable was linearized by calculating the middle point of the amounts, and was standardized. Therefore, z-scores were used in further data analysis.

*Perceived Relative Material Wealth* is assessed using the question and response options derived from national surveys in Hungary, such as the National Survey on Addiction Problems in Hungary (OLAAP) [34] and the European School Survey Project on Alcohol and Other Drugs (ESPAD) [35]. Participants are asked to indicate the extent to which they perceive their family’s current material wealth compared to others. Participants are also asked to rate their material wealth when the participant was a child. The response format is a seven-point Likert scale ranging from 1 (highly among the best) to 7 (among the worst). Therefore, lower scores indicate higher perceived material wealth.

*Vocabulary Test* (VOCAB) consists of 30 stimulus words randomly selected from an online quiz prepared by *Encyclopedia Britannica* [36]. Each stimulus word is followed by four possible one-word definitions, of which one is correct. Examples of stimulus words and their correct definitions that we used are: vain-conceited, amorphous-shapeless, and elucidate-explain. We pilot tested VOCAB with a sample of 35 American university students, and based on the results (mean correct = 12.54, SD = 4.70) we substituted three new words (futile, condone, jargon) for the three most frequently missed words on the pilot version, and retested with a larger and better educated sample. The result was a mean of 23.10 and an SD of 6.11. We replicated the pilot test in a Hungarian sample of adults (*N* = 76; 77.6% female, *M*_*age*_ = 26.5 years, *SD* = 5.5). Participants were highly educated (63.2% had college degree or higher, 34.2% secondary school certificate, and only 2.6% completed eight classes or less). Items were translated and back-translated following the protocol by [37]. The mean of correct answers was 27.96 (*SD* = 1.75). Six items were answered correctly by all of the participants. Participants spent an average of 163.76 s (about 2 min and 44 s) completing the test (5.46 s/item). The time limit was set to 4 min. In order to decrease the ceiling effect, the six items that could be answered correctly by all of the participants were deleted. Therefore, 24 items remained for which an average completion time was estimated and determined at 131 s. Based on this, a new time constraint (2 min) was set for the final data collection. The purpose of the Vocabulary test was to serve as a brief measure of crystallized intelligence. Previous studies have used a vocabulary test as a brief substitute for a battery of crystallized measures [38, 39] because vocabulary scores correlate highly with several other crystallized measures, but minimally with measures of fluid intelligence [24].

The *Short Digit Symbol Test* (SDST) is a 30-item subtest modeled after the *Digit Symbol Test* found on the *Multidimensional Aptitude Battery-Form L* [40]. The SDST is similar in that it contains nine symbols, each corresponding to digits one through nine, and five possible answers, labeled “A” through “E.” Another similarity is that the initial items are easy and gradually become more difficult. For example, Item one on both subtests has only one pairing of a symbol with a digit, items 2, and 3 pair two symbols with two digits, and items 4, 5, 6, and 7 pair three symbols with three digits. Yet another similarity is that the correct answer is evenly spread across all five possible answers. For the more difficult items, such as item 30, which has eight pairs of symbols with eight digits on both subtests, the correct answer choice is deliberately made more difficult by making one or more incorrect answers similar to the correct one. Dissimilarities include the fact that the *Digit Symbol Test* contains 35 items, compared to 30 on the SDST. The *Digit Symbol Test* should be slightly more difficult, because items 31–35 all contain nine symbols, whereas the SDST never contains more than eight. To adjust for this difference, time allotted for the SDST is six minutes as compared to seven minutes allotted for the *Digit Symbol Test*. Also, the symbols correspond to different numbers. For example, the + symbol corresponds to 6 on the *Digit Symbol Test* but the + symbol corresponds to 7 on the SDST. The object of both tests is to get as many items correct as possible within the allotted time. To do so, it requires the test-taker to remember which symbol corresponds to which digit. Constantly having to look at the box of symbols and digits at the top of each page is time-consuming, making it more difficult to finish within the allotted time. The task is made more difficult by presenting one or more incorrect answers on each of the items that are similar to the correct answer. A pilot test was conducted with the participation of 36 Hungarian adults (72.2% female, *M*_*age*_ = 27.3 years, SD = 7.9). Participants were again highly educated (50.0% had college degree or higher, 47.2% secondary school certificate, and 2.8% completed eight classes or less). The average of correct answers was 18.4 (*SD* = 5.8) using a time constraint of 6 min. Provided that the range of correct answers (from 7 to 30) was wide, the time limit of 6 min was set for the final data collection.

### Statistical analysis

The measures described above were assembled in several different orders to reduce the likelihood of a systematic order effect. Statistical analysis was carried out using SPSS version 20 (IBM SPSS Inc., Chicago, Illinois). First, zero-order correlations were computed to explore the associations between cognitive ability test scores, and celebrity worship scores (Hypothesis 1), as well as several variables expected to be related to either cognitive ability or celebrity worship. Second, partial correlation was performed to determine if the cognitive test scores would be negatively related to total celebrity worship scores (Hypothesis 2) even after controlling for gender, age, educational level, self-esteem, current family income, current material wealth, and perceived material wealth as a child. The VOCAB and the SDST test scores were combined in a composite z-score for this analysis. In the following step, the contribution of variables to cognitive performance was explored using multiple linear regressions (RQ1). In order to investigate the predictive power of each variable, univariate linear regressions were also conducted in which the independent variables were entered separately.

## Results

### Associations between celebrity worship, material wealth and cognitive ability

To test H1, associations between study variables were investigated. According to the results (see Table 1), higher scores on the three dimensions of celebrity worship were consistently associated with lower performance on the two cognitive ability tests (i.e., the vocabulary test and the digit symbol test), although these associations generally were weak (*r*s were between − 0.05 and − 0.12, respectively). Therefore, H1 was supported.

**Table 1:** Zero-order correlations among study-variables (*N* = 1763)

	Mean (SD)	1	2	3	4	5	6	7	8	9	10	11	12
1. Age	37.22 (12.06)	–											
2. Educational level	–	0.22***	–										
3. CAS Entertainment–Social subscale (range: 10–50)	22.46 (7.20)	− 0.12***	− 0.10***	–									
4. CAS Intense–Personal subscale (range: 9–45)	14.57 (5.71)	− 0.08***	− 0.10***	0.73***	–								
5. CAS Borderline–Pathological subscale (range: 4–20)	6.36 (2.48)	− 0.16***	− 0.08***	0.65***	0.63***	–							
6. CAS Total (range: 23–115)	43.38 (13.85)	− 0.12***	− 0.10***	0.94***	0.91***	0.78***	–						
7. Self-esteem (range: 10–40)	29.56 (5.85)	0.23***	0.16***	− 0.13***	− 0.14***	− 0.19***	− 0.16***	–					
8. Current family income (z-score)	–	0.12***	0.22***	− 0.10***	− 0.12***	− 0.09***	− 0.12***	0.22***	–				
9. Material wealth (current) (range: 1–7)	3.12 (1.00)	0.05*	− 0.16***	0.03	0.06*	0.003	0.04	− 0.25***	− 0.48***	–			
10. Material wealth (child) (range: 1–7)	3.68 (1.19)	0.16***	− 0.01	− 0.04	− 0.04	− 0.07**	− 0.05	− 0.06*	− 0.03	0.29***	–		
11. VOCAB (range: 0–24)	22.09 (1.55)	0.07**	0.17***	− 0.06*	− 0.10***	− 0.09***	− 0.09***	0.02	0.10***	− 0.04	0.05*	–	
12. SDST (range: 0–30)	14.77 (8.05)	− 0.20***	0.12***	− 0.06**	− 0.08**	− 0.05*	− 0.08**	0.02	0.05*	− 0.07**	− 0.04	0.10***	–
13. Cognitive tests (z-score)	–	− 0.09***	0.18***	− 0.08**	− 0.12***	− 0.09***	− 0.11***	0.03	0.11***	− 0.07**	0.006	0.74***	0.74***

***p < 0.001; **p < 0.01; *p < 0.05

*CAS* celebrity attitude scale, *VOCAB* vocabulary test, *SDST* short digit symbol test

Educational level was coded as 1 = “8 classes of less”, 2 = “Secondary school certificate” and 3 = “College degree or higher”, and Spearman rank-order correlations were calculated for this variable, while Pearson-correlations were calculated for all other variables. Z-scores were used for the linearized variable of current family income

Cognitive tests reflect a composite z-score derived from z-scores of VOCAB and SDST

Consistent with H2, partial correlations confirmed the weak, negative relationship among celebrity worship and cognitive skills in all aspects except for the relationship between the vocabulary test and the Entertainment–Social dimension of celebrity worship, which was not significant (see Table 2). Demographic characteristics, self-esteem, current income and material wealth were control variables in this analysis.

**Table 2:** Partial correlations among celebrity worship and cognitive skills (*N* = 1763)

	1	2	3	4	5	6
1. CAS Total (range: 23–115)	–					
2. CAS Entertainment–Social subscale (range: 10–50)	0.94***	–				
3. CAS Intense–Personal subscale (range: 9–45)	0.90***	0.73***	–			
4. CAS Borderline–Pathological subscale (range: 4–20)	0.77***	0.64***	0.61***	–		
5. VOCAB (range: 0–24)	− 0.07**	− 0.04	− 0.10***	− 0.07**	–	
6. SDST (range: 0–30)	− 0.08**	− 0.07*	− 0.07**	− 0.06*	0.10***	–
7. Cognitive tests (z-score)	− 0.10***	− 0.07**	− 0.11***	− 0.09**	0.75***	0.74***

***p < 0.001; **p < 0.01; *p < 0.05

*CAS* celebrity attitude scale, *VOCAB* vocabulary test, *SDST* short digit symbol test

The variable “cognitive tests” reflect a composite z-score combined from the z-scores of VOCAB and SDST

Control variables were gender, age, educational level, current family income, current and childhood material wealth and self-esteem

### The associations of cognitive performance

In the second step, linear regressions were conducted to explore the most influential predictors of cognitive performance (see Table 3). The model of cognitive tests (F_(8, 1536)_ = 14.58; *p* < 0.001), VOCAB (F_(8, 1536)_ = 8.09; *p* < 0.001) and SDST (F_(8, 1536)_ = 17.58; *p* < 0.001) were all significant.

**Table 3:** Linear regression models of cognitive performance (*N* = 1763)

Independent variables	Dependent variables β (SE)		
	Cognitive tests	VOCAB	SDST
Gender	0.02 (0.04)	0.07 (0.12)**	− 0.04 (0.42)
Age	− 0.15 (0.002)***	0.02 (0.005)	− 0.25 (0.02)***
Educational level	0.19 (0.04)***	0.12 (0.12)***	0.17 (0.46)***
Self-esteem	0.005 (0.003)	− 0.01 (0.01)	0.02 (0.04)
Current family income	0.07 (0.02)*	0.07 (0.06)*	0.03 (0.23)
Material wealth (current)	− 0.01 (0.02)	− 0.01 (0.06)	− 0.009 (0.24)
Material wealth (child)	0.03 (0.02)	0.05 (0.05)	− 0.01 (0.17)
Celebrity worship	− 0.10 (0.01)***	− 0.07 (0.004)**	− 0.07 (0.01)**
R^2^	6.6%	3.5%	7.9%

***p < 0.001; **p < 0.01; *p < 0.05

β (SE) = standardized coefficient and its standard error

*VOCAB* vocabulary test, *SDST* short digit symbol test

Cognitive tests represent the composite score derived from the z-scores of the VOCAB and the SDST

Gender was coded as 1 = “male” and 2 = “female”

Educational level was coded as 0 = “less than college degree” and 1 = “college degree or higher”

For the linearized variable of current family income, z-scores were used

It was found that individuals with lower educational level and higher levels of celebrity worship consistently performed poorer on the cognitive tests. Furthermore, females yielded higher scores on the VOCAB test, while younger individuals had higher scores on the SDST test. Additionally, a weak association was found between current family income and individuals’ performance on the VOCAB test, indicating that those who reported higher income had slightly higher scores on the SDST test than individuals with lower family income. However, these associations were generally weak, and the variables explained only a small proportion of the total variance of cognitive performance (3.5% and 7.9%). Similar associations were found when the predictive power of celebrity worship dimensions was explored using the same model structure (see Additional file 1: Appendix 1).

Univariate regressions confirmed these associations (see Table 4). Moreover, current material wealth and relative lack of childhood material wealth were associated with slightly higher performance on the VOCAB test. In addition, higher current family income was associated with slightly higher performance on both cognitive tests. It was also found that increasing age was related to higher performance on the VOCAB test. Univariate regressions also indicated that celebrity worship remained a consistent predictor of poor performance on the cognitive tests. Moreover, the three dimensions of celebrity worship were consistently associated with poorer cognitive performance on the tests (see Additional file 2: Appendix 2). However, these associations were again weak, and the proportion of explained variance was small (below 5% for all variables).

**Table 4:** Univariate regression models of cognitive performance (*N* = 1763)

Independent variables	Dependent variables β (SE)					
	Cognitive tests	R^2^ (%)	VOCAB	R^2^ (%)	SDST	R^2^ (%)
Gender	0.02 (0.04)	0	0.07 (0.11)**	0.5	− 0.04 (0.41)	0
Age	− 0.09 (0.001)***	0.8	0.07 (0.004)**	0.4	− 0.20 (0.02)***	4.1
Educational level	0.18 (0.04)***	3.2	0.15 (0.11)***	2.3	0.12 (0.42)***	1.3
Self-esteem	0.03 (0.003)	0	0.02 (0.009)	0	0.02 (0.03)	0
Current family income	0.11 (0.02)***	1.0	0.10 (0.05)***	1.0	0.05 (0.20)*	0.2
Material wealth (current)	− 0.07 (0.02)**	0.5	− 0.04 (0.05)	0	− 0.07 (0.19)**	0.4
Material wealth (child)	0.006 (0.02)	0	0.05 (0.04)*	0	− 0.04 (0.16)	0
Celebrity worship	− 0.11 (0.001)***	1.1	− 0.09 (0.004)***	0.7	− 0.08 (0.01)**	0.5

***p < 0.001; **p < 0.01; *p < 0.05

β (SE) = standardized coefficient and its standard error

*VOCAB* vocabulary test, *SDST* short digit symbol test

Cognitive tests represent the composite score derived from the z-scores of the VOCAB and the SDST

Gender was coded as 1 = “male” and 2 = “female”

Educational level was coded as 0 = “less than college degree” and 1 = “college degree or higher”

For the linearized variable of current family income, z-scores were used

## Discussion

There have been mixed findings in the literature regarding the association between celebrity worship and cognitive skills. This study endeavored to investigate this association using a relatively large sample of Hungarian adults, and explore the nature of this relationship by involving some relevant demographic, socioeconomic and psychological variables (e.g., educational level, material wealth, self-esteem) in the analysis. As a main result, it was found that the direct association between celebrity worship and poorer cognitive performance was weak but consistent, even after controlling for demographic and socioeconomic variables such as educational level and material wealth. This result may suggest that deeper involvement with a celebrity may be directly associated with poorer performance in tasks requiring attention and focus, which may be explained by the cognitive effort put into maintaining the absorption and the one-sided emotional bond with an admired celebrity. This process can possibly challenge the cognitive capacity of fans and limit their attention skills and effort invested in other activities that are unrelated to the admired celebrity.

### Hypotheses and theoretical contextualization

Consistent with the first hypothesis and previous research [1, 23], a weak, negative relationship was found between *Celebrity Attitude Scale* (CAS) scores and scores on the measure of crystallized intelligence (i.e., the vocabulary test) and fluid intelligence (i.e., the short digit symbol test). Previous studies concluded that there was a weak relationship between celebrity worship and some cognitive skills (e.g., spatial ability, critical thinking), while this association could not be observed in relation to other skills (e.g., problem solving, intellectual flexibility) [1, 5]. Provided that both components of Cattell’s model were associated with celebrity worship in the present study, this result suggests that an excessive admiration toward a celebrity may interfere with cognitive performance on a more global level. However, further studies are needed to confirm this finding. These studies should use a more comprehensive theoretical framework to provide a more nuanced picture of the association of celebrity worship with cognitive skills.Supporting the second hypothesis, a direct association was found between celebrity worship and performance on the two cognitive tests even after controlling for demographic characteristics, current income, material wealth and self-esteem. This result suggests that celebrity worship has a direct association with poorer cognitive performance on tasks targeting crystallized and fluid intelligence, and this association cannot be attributed solely to educational level or sociodemographic characteristics of fans.

In the following step, the unique contribution of celebrity worship to the explanation of lower performance on the cognitive tests was explored using regression models. These models indicated that the strongest predictor of lower cognitive performance on the vocabulary test was lower educational level, followed by lower current family income and higher celebrity worship levels. Furthermore, male participants also performed poorer than female participants on the vocabulary test. Consistent with these results, the strongest predictors of lower performance on the digit symbol test were lower educational level, older age and higher celebrity worship levels. However, the explanatory power of these factors were particularly limited (below 5%), indicating that the contribution of demographic characteristics and celebrity worship to lower performance on these cognitive tests is negligible.

The present findings regarding the weak, negative association between celebrity worship and cognitive performance are in line with some previous studies [2, 15, 23]. McCutcheon et al. [1] suggested that a possible explanation for this association may be that individuals with higher levels of cognitive skills are more likely to understand the marketing strategies behind a famous person, which may prevent them from developing a strong emotional bond with the celebrity, who may be perceived as a product of the media. Another possible explanation for this association could be that celebrity worship has been associated with addictive behaviors such as gambling addiction, compulsive buying and problematic Internet and social media use (see [41] for a review). A core feature of behavioral addictions is the decreased level of cognitive flexibility and performance [42]. Furthermore, celebrity worship has been associated with increased fantasy proneness [43] and maladaptive daydreaming [7], which can interfere with academic performance and cognitive control functions [44]. Indeed, it can be assumed that an excessive involvement with a celebrity may be demanding in terms of cognitive effort, since the maintenance of this one-sided emotional bond with the favorite celebrity—that may become dominant in the life of some fans—requires attention. In line with this assumption, the present findings suggest that celebrity worship as an excessive behavior has a direct association with poorer performance on cognitive tests that measure crystallized and fluid intelligence, which cannot be accounted for by specific demographic characteristics, material wealth or self-esteem. However, the explanatory power of celebrity worship in poorer performance was particularly limited across the two tests, which indicates that admiration toward a famous person is not a powerful predictor of impaired cognitive functioning. This result is in line with a recent suggestion by McCutcheon et al. [5] who proposed that celebrity worship may not play an influential role in attention deficit and other problems in cognitive functioning, although there is a weak, consistent relationship between these two constructs.

### Limitations

In spite of efforts to ensure equivalent meanings, it is possible that the translation from one language to another changed the meaning of some scale items. In spite of that, our results were generally consistent with results obtained in studies conducted in English-speaking countries. Furthermore, it worth mentioning that cross-sectional study design was applied. Therefore, it is not possible to draw conclusions regarding the direction of the associations between variables in this study. Underlying mechanisms and causes of the associations cannot be identified, either which limits the understanding of the nature of the association between the study variables. Besides, the use of self-report measures can possibly bias the results due to social desirability or memory recall deficiencies. Another limitation is the limited explanatory power of demographics, social and psychological variables in cognitive performance. Despite identifying some significant predictors, educational level, current family income and celebrity worship explained only a small proportion of the total variance of cognitive performance (below 5%). Based on the weak correlations between study variables, health care professionals should act with caution when designing interventions and implementing specific elements based upon the current findings (e.g., the efficacy of intervention may underperform the initial expectations). Future studies should extend the scope of investigation to other possibly relevant factors that can influence cognitive performance (e.g., impulsivity, daydreaming).

## Conclusion

Despite the limitations, this study indicated a weak, negative association between celebrity worship and cognitive performance even after controlling for some relevant demographic, socioeconomic and psychological factors. These results align with previous findings on addictive behaviors, which suggest that excessive behaviors can impair cognitive functioning due to the increased focus and energy invested in the behavior that dominates the person’s life (i.e., celebrity worship in this case). However, the explanatory power of celebrity worship on lower cognitive performance was limited, suggesting that the admiration toward a celebrity is not a prominent predictor of poorer cognitive skills, although there is a consistent, weak relationship between the two constructs. Based upon this finding, celebrity worship can be regarded as one contributing factor that may alter cognitive performance beside—and independent from—education, age and material wealth, although other factors may be stronger predictors of cognitive performance (e.g., attitudes toward performance, school/work performance, perfectionism). This study extended previous research by applying Cattell’s theoretical model to investigate the association between celebrity worship and cognitive skills. Furthermore, this study was among the first that explored the contribution of celebrity worship and socioeconomic factors to cognitive performance in one comprehensive model. In comparison with prior studies, this study used a relatively large sample of Hungarian adults, which allowed for the investigation of these associations in a culturally different context. Based upon the findings, it may be concluded that cognitive performance is slightly altered when higher celebrity worship levels are expressed, although cognitive skills seem to be largely independent of celebrity admiration. This conclusion aligns with the recommendation by McCutcheon et al. [5], who proposed that the association between these two constructs may not be so robust as suggested by the first studies of this relationship. Future research should explore the directionality of this association. It is possible that individuals with stronger cognitive abilities are less likely to express higher celebrity worship levels because they can recognize the marketing strategies behind a celebrity, as suggested by McCutcheon et al. [1], but it is also possible that the cognitive effort put into maintaining the absorption in a celebrity may interfere with other tasks that require attention and focus. Longitudinal research is needed to draw clearer conclusions about causality. Furthermore, these studies should also explore a broader range of variables that may contribute to cognitive performance (e.g., attitudes toward performance, perfectionism) in order to extend knowledge on the contribution of celebrity worship to cognitive skills. The exploration of such factors in a more comprehensive model could possibly contribute to a better understanding of the variability of cognitive performance among fans with different levels of dedication for their favorite celebrity.

## Supplementary Information

**Additional file 1: Appendix 1.** Linear regression models with celebrity worship dimensions predicting cognitive performance (N = 1763). **Note:** ***p < 0.001; **p < 0.01; *p < 0.05; β (SE) = standardized coefficient and its standard error; VOCAB = Vocabulary Test; SDST = Short Digit Symbol Test; Cognitive tests represent the composite score from the z-scores of the VOCAB and the SDST. Gender was coded as 1 = “male” and 2 = “female”; Educational level was coded as 0 = “less than college degree” and 1 = “college degree or higher” Z-score was used for the linearized variable of current family income. The models using cognitive tests (F10,1534 = 12.27; p < 0.001), VOCAB (F10,1534 = 7.42; p < 0.001) and SDST (F10,1534 = 14.08; p < 0.001) as an outcome variable were all significant.

**Additional file 2: Appendix 2.** Univariate regression models with celebrity worship dimensions predicting cognitive performance (N = 1763). **Note:** ***p < 0.001; **p < 0.01; *p < 0.05; β (SE) = standardized coefficient and its standard error; CAS = Celebrity Attitude Scale; VOCAB = Vocabulary Test; SDST = Short Digit Symbol Test; Cognitive tests represent the composite score calculated from the z-scores of the VOCAB and the SDST. Gender was coded as 1 = “male” and 2 = “female”; Educational level was coded as 0 = “less than college degree” and 1 = “college degree or higher” Z-score was used for the linearized variable of current family income.

## Data Availability

The datasets used and/or analysed during the current study available from the corresponding author on reasonable request.
