# Supplementary material for: Celebrity worship and cognitive skills revisited: applying Cattell’s two-factor theory of intelligence in a cross-sectional study
Source: BMC Psychol. 2021 Nov 8;9:174. doi: 10.1186/s40359-021-00679-3 (PMC8574017; doi:10.1186/s40359-021-00679-3)
Supplement: Supplementary file 1 — Additional file 1: Appendix 1. Linear regression models with celebrity worship dimensions predicting cognitive performance (N = 1763). Note: ***p < 0.001; **p < 0.01; *p < 0.05; β (SE) = standardized coefficient and its standard error; VOCAB = Vocabulary Test; SDST = Short Digit Symbol Test; Cognitive tests represent the composite score from the z-scores of the VOCAB and the SDST. Gender was coded as 1 = “male” and 2 = “female”; Educational level was coded as 0 = “less than college degree” and 1 = “college degree or higher” Z-score was used for the linearized variable of current family income. The models using cognitive tests (F10,1534 = 12.27; p < 0.001), VOCAB (F10,1534 = 7.42; p < 0.001) and SDST (F10,1534 = 14.08; p < 0.001) as an outcome variable were all significant. [file 40359_2021_679_MOESM1_ESM.docx]

Appendix 1. Linear regression models with celebrity worship dimensions predicting cognitive performance (*N* = 1,763)

| Predictor variables | Outcome variables β (SE) | | |
| --- | --- | --- | --- |
|  | Cognitive tests | VOCAB | SDST |
| Gender | 0.02 (0.04) | 0.07 (0.12)^**^ | -0.04 (0.43) |
| Age | -0.15 (0.002)^***^ | 0.03 (0.005) | -0.25 (0.02)^***^ |
| Educational level | 0.19 (0.04)^***^ | 0.12 (0.12)^***^ | 0.17 (0.46)^***^ |
| Self-esteem | 0.005 (0.003) | -0.02 (0.01) | 0.02 (0.04) |
| Current family income | 0.07 (0.02)^*^ | 0.07 (0.06)^*^ | 0.03 (0.23) |
| Material wealth (current) | -0.01 (0.02) | -0.01 (0.06) | -0.009 (0.24) |
| Material wealth (child) | 0.02 (0.02) | 0.05 (0.05) | -0.01 (0.17) |
| CAS Entertainment–Social | 0.04 (0.004) | 0.07 (0.01) | -0.02 (0.04) |
| CAS Intense–Personal | -0.11 (0.005)^**^ | -0.13 (0.01)^**^ | -0.04 (0.05) |
| CAS Borderline–Pathological | -0.04 (0.01) | -0.04 (0.03) | -0.02 (0.11) |
| R^2^ | 6.8% | 4.0% | 7.8% |

Notes: ^***^*p* < 0.001; ^**^*p* < 0.01; ^*^*p* < 0.05

β (SE) *=* standardized coefficient and its standard error

VOCAB = Vocabulary Test; SDST = Short Digit Symbol Test

Cognitive tests represent the composite score from the z-scores of the VOCAB and the SDST.

Gender was coded as 1 = “male” and 2 = “female”

Educational level was coded as 0 = “less than college degree” and 1 = “college degree or higher”

Z-score was used for the linearized variable of current family income.

The models using cognitive tests (F_10,1534_ = 12.27; p < 0.001), VOCAB (F_10,1534_ = 7.42; p < 0.001) and SDST (F_10,1534_ = 14.08; p < 0.001) as an outcome variable were all significant.
