# Supplementary material for: Celebrity worship and cognitive skills revisited: applying Cattell’s two-factor theory of intelligence in a cross-sectional study
Source: BMC Psychol. 2021 Nov 8;9:174. doi: 10.1186/s40359-021-00679-3 (PMC8574017; doi:10.1186/s40359-021-00679-3)
Supplement: Supplementary file 2 — Additional file 2: Appendix 2. Univariate regression models with celebrity worship dimensions predicting cognitive performance (N = 1763). Note: ***p < 0.001; **p < 0.01; *p < 0.05; β (SE) = standardized coefficient and its standard error; CAS = Celebrity Attitude Scale; VOCAB = Vocabulary Test; SDST = Short Digit Symbol Test; Cognitive tests represent the composite score calculated from the z-scores of the VOCAB and the SDST. Gender was coded as 1 = “male” and 2 = “female”; Educational level was coded as 0 = “less than college degree” and 1 = “college degree or higher” Z-score was used for the linearized variable of current family income. [file 40359_2021_679_MOESM2_ESM.docx]

Appendix 2. Univariate regression models with celebrity worship dimensions predicting cognitive performance (*N* = 1,763)

| Predictor variables | Outcome variables β (SE) | | | | | |
| --- | --- | --- | --- | --- | --- | --- |
|  | Cognitive tests | R^2^ | VOCAB | R^2^ | SDST | R^2^ |
| Gender | 0.02 (0.04) | 0% | 0.07 (0.11)^**^ | 0.5% | -0.04 (0.41) | 0% |
| Age | -0.09 (0.001)^***^ | 0.8% | 0.07 (0.004)^**^ | 0.4% | -0.20 (0.02)^***^ | 4.1% |
| Educational level | 0.18 (0.04)^***^ | 3.2% | 0.15 (0.11)^***^ | 2.3% | 0.12 (0.42)^***^ | 1.3% |
| Self-esteem | 0.03 (0.003) | 0% | 0.02 (0.009) | 0% | 0.02 (0.03) | 0% |
| Current family income | 0.11 (0.02)^***^ | 1.0% | 0.10 (0.05)^***^ | 1.0% | 0.05 (0.20)^*^ | 0.2% |
| Material wealth (current) | -0.07 (0.02)^**^ | 0.5% | -0.04 (0.05) | 0% | -0.07 (0.19)^**^ | 0.4% |
| Material wealth (child) | 0.006 (0.02) | 0% | 0.05 (0.04)^*^ | 0% | -0.04 (0.16) | 0% |
| CAS Entertainment–Social | -0.08 (0.002)^**^ | 0.7% | -0.06 (0.007)^*^ | 0.3% | -0.06 (0.03)^**^ | 0.4% |
| CAS Intense–Personal | -0.12 (0.003)^***^ | 1.4% | -0.10 (0.009)^***^ | 0.9% | -0.08 (0.03)^**^ | 0.6% |
| CAS Borderline–Pathological | -0.09 (0.007)^***^ | 0.8% | -0.09 (0.02)^***^ | 0.7% | -0.05 (0.08)^*^ | 0.2% |

Notes: ^***^*p* < 0.001; ^**^*p* < 0.01; ^*^*p* < 0.05

β (SE) *=* standardized coefficient and its standard error

CAS = Celebrity Attitude Scale; VOCAB = Vocabulary Test; SDST = Short Digit Symbol Test

Cognitive tests represent the composite score calculated from the z-scores of the VOCAB and the SDST.

Gender was coded as 1 = “male” and 2 = “female”

Educational level was coded as 0 = “less than college degree” and 1 = “college degree or higher”

Z-score was used for the linearized variable of current family income.
